# Limonoids from *Melia azedarach* Fruits as Inhibitors of Flaviviruses and *Mycobacterium tubercolosis*

**DOI:** 10.1371/journal.pone.0141272

**Published:** 2015-10-20

**Authors:** Giuseppina Sanna, Silvia Madeddu, Gabriele Giliberti, Nikoletta G. Ntalli, Filippo Cottiglia, Alessandro De Logu, Emanuela Agus, Pierluigi Caboni

**Affiliations:** 1 Department of Biomedical Sciences, Section of Microbiology and Virology, University of Cagliari, Italy; 2 Department of Life and Environmental Sciences, High Resolution Mass Spectrometry Laboratory, University of Cagliari, Italy; 3 Department of Life and Environmental Sciences, Section of Medical Microbiology, University of Cagliari, Italy; University of Washington, UNITED STATES

## Abstract

The biological diversity of nature is the source of a wide range of bioactive molecules. The natural products, either as pure compounds or as standardized plant extracts, have been a successful source of inspiration for the development of new drugs. The present work was carried out to investigate the cytotoxicity, antiviral and antimycobacterial activity of the methanol extract and of four identified limonoids from the fruits of *Melia azedarach* (Meliaceae). The extract and purified limonoids were tested in cell-based assays for antiviral activity against representatives of ssRNA, dsRNA and dsDNA viruses and against *Mycobacterium tuberculosis*. Very interestingly, 3-*α*-tigloyl-melianol and melianone showed a potent antiviral activity (EC_50_ in the range of 3–11μM) against three important human pathogens, belonging to *Flaviviridae* family, West Nile virus, Dengue virus and Yellow Fever virus. Mode of action studies demonstrated that title compounds were inhibitors of West Nile virus only when added during the infection, acting as inhibitors of the entry or of a very early event of life cycle. Furthermore, 3-*α*-tigloyl-melianol and methyl kulonate showed interesting antimycobacterial activity (with MIC values of 29 and 70 μM respectively). The limonoids are typically lipophilic compounds present in the fruits of *Melia azeradach*. They are known as cytotoxic compounds against different cancer cell lines, while their potential as antiviral and antibacterial was poorly investigated. Our studies show that they may serve as a good starting point for the development of novel drugs for the treatment of infections by *Flaviviruses* and *Mycobacterium tuberculosis*, for which there is a continued need.

## Introduction

*Melia azedarach*, commonly known as chinaberry, is a species of the botanical family Meliaceae exhibiting a range of biological activities. Crude and petroleum ether extracts of *M*. *azedarach* leaves and bark recently were found of significant activity against *Streptococcus mutans* [1]. The chloroform bark extract was active against *Enterobacter aerogenes* and *Proteus mirabilis*, while the ethyl acetate bark extract exhibited activity against *Pseudomonas aeruginosa* [2]. Extracts as well as ingredients purified from various parts of *M*. *azedarach* are also reported to exhibit activity against human and animal parasites [3–5], and to possess antioxidant and antifungal activities [6]. 1-Cinnamoyl-3,11 dihydroxymeliacarpinin, a limonoid isolated form *M*. *azedarach*, was found to inhibit herpes simplex virus [7,8].

The increasing interest in chinaberry’s properties is in fact demonstrated by the recent attempts of its large-scale production by in vitro regeneration [9,10]. Chinaberry active compounds are typically lipophilic limonoids, found in fruits [11]. Limonoids from *M*. *azedarach* are described as significantly cytotoxic against different cancer cell lines [12–17].

To date, limonoids of other botanical origin have been described as active against HIV-1 [18–19], dengue virus [20,21], respiratory syncytial virus [22] and herpes simplex virus [23]. This is the first report on the antiviral activity of limonoids from *M*. *azedarach*; they showed activity against three important human pathogens of Flavivirus genus: Dengue virus (DENV), West Nile virus (WNV) and Yellow fever virus (YFV). Furthermore, this is the first report on antimycobacterial activity of these four tirucallanes except for 21-β-acetoxy-melianone that has been already reported to act against *M*. *tuberculosis* with a MIC value of 16 μg/ml [24].

Flaviviruses are the most prevalent arthropod-borne viruses worldwide and most of them are transmitted to vertebrates by mosquitoes or ticks [25], causing disease and mortality. Their infections are continuously re-emerging throughout the world [26,27]. Although effective vaccines are in use for some of them like YFV [28], despite their clinical impact there is no specific human antiviral therapy available to treat infection with any of the flaviviruses. Therefore, there is a continued need for novel drugs and therapies [29].

In our previous studies, we found that *M*. *azedarach* tirucallane-type triterpenes, namely 3-α-tigloyl-melianol, melianone, 21-β-acetoxy-melianone and methyl kulonate exhibit cytotoxicity towards the human lung adenocarcinoma epithelial cell line A549 [30].

In the present investigation, we report on: 1) the evaluation of antiviral properties of selected limonoids, as well as of the methanolic extract from *M*. *azedarach*, in an antiviral screening against HIV-1 and other RNA and DNA viruses belonging to different families, including several important human pathogens; 2) the activity of selected limonoids against *M*. *tuberculosis*.

## Materials and Methods

### Chemicals

Methanol was of high performance liquid chromatography (HPLC) grade (Baker, Milan, Italy); hexane, ethyl acetate, dichloromethane and ethanol were of gas chromatography grade purchased from Baker (Milan, Italy); vanillin, and sulphuric acid were obtained from Carlo Erba (Milan, Italy); and the water was distilled and filtered through a Milli-Q apparatus (Millipore, Milan, Italy) before use. The aluminium TLC plates 20 x 20 cm silica gel 60 F254, 0.25 mm were purchased from Merck, while the silica gel grade 70–230 mesh, 60 A was purchased from Sigma Aldrich (Milan, Italy). The chromogenic solution used for the revelation of the compounds on TLC was composed of: a) ethanol-sulfuric acid (95:5; v/v) and b) ethanol-vanillin (99:1; v/w).

#### Extraction and purification of limonoids from fruits

Ripe fruits of *M*. *azedarach* were collected at Volos, Greece in February 2013. No specific permission was required for this location, since that it is not an endangered or protected specie. Voucher specimens were deposited at the Department of Life and Environmental Sciences, University of Cagliari, Italy, for species identification. 100 gr of ripe and defatted chinaberry fruits were extracted and purified by column chromatography with a slight modification of the procedure previously reported by our laboratory [30]. Ripe fruits were placed in a sonicator for 30 min with methanol to yield 47 g of Melia methanol extract (MME), which was thereafter suspended in methanol-water (50:50; v/v) and partitioned with dichloromethane (DCM) to afford 6 gr of a limonoids fraction. Half the amount of the limonoids fraction (3 g) was then subjected to open column chromatography (CC; 60 x 4 cm) (silica gel, 300 g) and eluted successively with hexane/ethyl acetate (Hex/EtOAc) 9/1; and 7/3; v/v and finally Hex 100% affording in total 1000 fractions. Examination by TLC allowed homogeneous fractions to be pooled (571–750 and 941–1000), giving two major fractions. The 571–750 major fraction was re-purified in CC with DCM/EtOAc (9.25/0.75; v/v) and homogeneous fractions (26–30 and 31–5), checked in TLC, were pooled to yield methyl kulonate (19.1 mg) and 3-*α*-tigloyl-melianol (20.28 mg). The 941–1000 major fraction was re-purified in CC with Hex/EtOAc (8/2; v/v) and homogeneous fractions (115–150), checked in TLC, were pooled to yield melianone (26.5 mg). NMR spectra and LC-QTOF-MS spectra of selected compounds were in accordance with those described in the previous isolation and characterization [30].

### Antimycobacterial activity

The minimum inhibitory concentration (MIC) of the DCM extract of *M*. *azedarach*, isolated limonoids and technical azadirachtin were determined by the standard method of the double dilution in agar against *M*. *tuberculosis* H37Ra ATCC 25177. Isoniazid (Sigma-Aldrich, St. Louis, MO) was used as a chemical control. Briefly: 1 mL of Middlebrook 7H11 agar (Difco Laboratories, Detroit, MI) enriched with a supplement of oleic acid-albumine-dextrose-catalase (OADC) at 10%, containing decreasing concentrations (range between 256 and 0.25 μg/ml) of the test compounds in 24 well plates, was inoculated with 100 *μ*L of a suspension containing 1.5x10^5^ CFU/mL obtained by dilution of a bacterial suspension grown in Middlebrook 7H9 broth (Difco Laboratories, Detroit, MI) and incubated at 37°C in an atmosphere of 5% CO_2_. After 28 day of incubation was determined the MIC, defined as the minimum concentration of the test compounds that inhibits completely the growth of mycobacteria. Cell viability in the presence of the triterpenoids was determined on Vero cells.

### Antiviral activity

#### Cells and viruses

Cell lines were purchased from American Type Culture Collection (ATCC). Cell lines supporting the multiplication of RNA and DNA viruses were the following: CD4+ human T-cells containing an integrated HTLV-1 genome (MT-4); Madin Darby Bovine Kidney (MDBK) [ATCC CCL 22 (NBL-1) *Bos Taurus*]; Baby Hamster Kidney (BHK-21) [ATCC CCL 10 (C-13) *Mesocricetus auratus*] and Monkey kidney (Vero 76) [ATCC CRL 1587 *Cercopithecus Aethiops*]. Human Immunodeficiency Virus type-1 (HIV-1) III_B_ laboratory strain was obtained from the supernatant of the persistently infected H9/III_B_ cells (NIH 1983). Viruses representative of ssRNA+ were: i) *Flaviviridae*: yellow fever virus (YFV) [strain 17-D vaccine (Stamaril Pasteur J07B01)], bovine viral diarrhoea virus (BVDV) [strain NADL (ATCC VR-534)], west nile virus (WNV) [Clinical isolate], dengue virus (DENV-2) [Clinical isolate]; ii) *Picornaviridae*: human enterovirus B [coxsackie type B5 (CVB-5), strain Ohio-1 (ATCC VR-29)], and human enterovirus C [poliovirus type-1 (Sb-1), Sabin strain Chat (ATCC VR-1562)]. Viruses representative of ssRNA were: iii) *Paramyxoviridae*: human respiratory syncytial virus (RSV) [strain A2 (ATCC VR-1540)]; iv) *Rhabdoviridae*: vesicular stomatitis virus (VSV) [lab strain Indiana (ATCC VR 1540)]. The virus representative of dsRNA was: iv) *Reoviridae*: reovirus type-1 (Reo-1) [simian virus 12, strain 3651 (ATCC VR-214)]. DNA virus representatives were: v) *Poxviridae*: vaccinia virus (VV) [vaccine strain Elstree-Lister (ATCC VR-1549)]; vi) *Herpesviridae*: human herpes 1 (HSV-1) [strain KOS (ATCC VR-1493)].

#### Cytotoxicity assays

Exponentially growing MT-4 cells were seeded at an initial density of 4x10^5^ cells/ml in 96-well plates in RPMI-1640 medium, supplemented with 10% fetal bovine serum (FBS), 100 units/mL penicillin G and 100 μg/mL streptomycin. MDBK and BHK cells were seeded in 24-well plates at an initial density of 6x10^5^ and 1x10^6^ cells/mL, respectively, in Minimum Essential Medium with Earle’s salts (MEM-E), L-glutamine, 1mM sodium pyruvate and 25mg/L kanamycin, supplemented with 10% horse serum (MDBK) or 10% foetal bovine serum (FBS) (BHK). Vero-76 cells were seeded in 24-well plates at an initial density of 4x10^5^ cells/mL, in Dulbecco’s Modified Eagle Medium (D-MEM) with L-glutamine and 25mg/L kanamycin, supplemented with 10% FBS. Cell cultures were then incubated at 37°C in a humidified, 5% CO_2_ atmosphere, in the absence or presence of serial dilutions of test compounds. Cell viability was determined after 48–96 hrs at 37°C by MTT method for MT-4, MDBK and BHK [31]. Cell viability was determined after 48–96 hrs at 37°C by the crystal violet staining method for Vero-76.

#### Antiviral assays

Compound’s activity against HIV-1 was based on inhibition of virus-induced cytopathogenicity in exponentially growing MT-4 cell acutely infected with a multiplicity of infection (m.o.i.) of 0.01. Briefly, 50 μL of RPMI containing 1x10^4^ MT-4 cells were added to each well of flat-bottom microtitre trays, containing 50 μL of RPMI without or with serial dilutions of test samples. Then, 20 μL of a HIV-1 suspension containing 100 CCID_50_ were added. After a 4-day incubation at 37°C, cell viability was determined by the MTT method. Compound’s activity against DENV-2, WNV, YFV and Reo-1 was based on inhibition of virus-induced cytopathogenicity in BHK-21 cells acutely infected with a m.o.i. of 0.01. Compound’s activity against BVDV was based on inhibition of virus-induced cytopathogenicity in MDBK cells acutely infected with a m.o.i. of 0.01. Briefly, BHK and MDBK cells were seeded in 96-well plates at a density of 5x10^4^ and 3x10^4^ cells/well, respectively, and were allowed to form confluent monolayers by incubating overnight in growth medium at 37°C in a humidified CO_2_ (5%) atmosphere. Cell monolayers were then infected with 50 μL of a proper virus dilution in maintenance medium [MEM-Earl with L-glutamine, 1mM sodium pyruvate and 0.025g/L kanamycin, supplemented with 0.5% inactivated FBS]. At the same time, 50 μL of maintenance medium, without or with serial dilutions of test compounds, were added. After a 3-/4-day incubation at 37°C, cell viability was determined by the MTT method. Compound’s activity against WNV was also detected by plaque reduction assays in infected BHK-21 cell monolayers, similarly to as following described, but with serial dilutions of test compounds added also during the infection. Compound’s activity against CVB-5, Sb-1, VV, HSV-1 and RSV was determined by plaque reduction assays in infected cell monolayers, as described earlier [32]. Briefly, Vero-76 monolayers were infected for 2 hours with 250 μL of proper virus dilutions; following removal of unadsorbed virus, 500 μL of maintenance medium containing 0.75% methyl-cellulose, without or with serial dilutions of test compounds, were added. Cultures were incubated at 37°C for 2 (Sb-1 and VSV), 3 (CVB-5, VV and HSV-1) or 5 days (RSV) and then fixed with PBS containing 50% ethanol and 0.8% crystal violet, washed and air-dried. Plaques were then counted. The extent of cell growth/viability and viral multiplication, at each sample concentration tested, were expressed as percentage of untreated controls. Concentrations resulting in 50% inhibition (CC_50_ or EC_50_) were determined by linear regression analysis.

#### Yield Reduction Assay

BHK-21 cells were inoculated with WNV at a m.o.i. of 0.1 in maintenance medium and tested compounds at not cytotoxic concentrations. After a 2 hrs adsorption period at 37°C and 5% CO_2_, the inoculum was removed and replaced with fresh medium containing the same concentration of compounds. After 72 hrs at 37°C and 5% CO_2_ each sample was harvested and diluted with serial passages, starting from 10^−1^ up to 10^−8^. The titre of the serial dilutions of the virus-containing supernatant was determined by standard plaque reduction assay.

#### Time of drug addition

In time of drug addition experiment, BHK-21 cells were infected with WNV as previously described. Tested compounds, as well as the reference compound 2’-C-methylguanosine, were added at different times: i) added at 2 h prior to up to the infection (pretreatment) and then removed before the infection; ii) added at the time of infection; iii) added at 1, 2, 4 and 8h post infection. After a 3 day incubation at 37°C, cell viability was determined by the MTT method.

#### Virucidal activity assay

A WNV suspension containing 5x10^5^ PFU/ml was incubated with or without different concentrations of compounds for 45 min at 37°C. At the end of incubation, the residual infectivity was determined by plaque reduction assay in BHK-21 cells.

#### Adsorption assay

BHK cells grown in 6-well plates were infected with WNV, with a m.o.i. of 1, in the presence or absence of compounds. Multiwell were incubated for 0, 15, 30 and 60 min at 4°C. Medium containing unadsorbed virus was then removed, cells were washed twice with PBS and covered with MEM containing 1% methylcellulose. Plaques were counted after 3 days of incubation at 37°C.

## Results and Discussion

The lipophilic limonoids are the typically active compounds of *M*. *azedarach*. They are described as significantly active against different cancer cell lines, as discussed in Introduction. On the contrary, their antiviral potential is poorly investigated.

In our previous studies, we characterized four tirucallane-type triterpenes from the fruits of *M*. *azedarach*: 3-α-tigloyl-melianol, melianone, 21-β-acetoxy-melianone and methyl kulonate. The isolated compounds, as well as the methanolic extract, were tested in cell-based assays against human immunodeficiency virus type-1 (HIV-1) and representative members of several RNA and DNA virus families. Results are reported in Table 1; antiviral activities were not found against CVB-5, Sb-1, RSV, VSV and VV viruses and results are so not reported in the table. In order to be able to establish whether test derivatives have selective antiviral activity, their cytotoxicity was evaluated in parallel assays with uninfected cell lines.

**Table 1:** Cytotoxicity and antiviral activity of triterpenoids from fruits of *M*. *azedarach*.

Compounds	MT-4	HIV-1_IIIB_	MDBK	BVDV	BHK-21	YFV	Reo-1	Vero-76	HSV-1
	^a^CC_50_	^e^EC_50_	^b^CC_50_	^f^EC_50_	^c^CC_50_	^g^EC_50_	^h^EC_50_	^d^CC_50_	^i^EC_50_
2methyl kulonate	35	>35	85	>85	>100	>100	>100	100	>100
321-β-acetoxy-melianone	23	>23	22	>22	10	>10	>10	36	>36
43-α-tigloylmelianol	27	>27	37	>37	20	7.0	>20	100	>100
5melianone	>100	>100	16	>16	50	3.0	>50	51	20
methanolic extract (μg/ml)	14	>14	>100	>100	53	>53	>53	>100	>100
*Ref*. *compounds*									
Efavirenz	40	0.002							
2’-C-methyl-guanosine			>100	2.0	80	1.4	1.0		
Acycloguanosine								>100	3.0

Data represent mean values of three independent determinations. The variation among them was less than 15% respect to the mean value.

^a-d^ Compound concentration (μM) required to reduce the proliferation of mock-infected MT-4^(a)^, MDBK^(b)^, BHK-21^(c)^ and Vero-76^(d)^ cells by 50%, as determined by the MTT method.

^e^ Compound concentration (μM) required to achieve 50% protection of MT-4 cells from HIV-1 induced cytopathogenicity, as determined by the MTT method.

^f^ Compound concentration (μM) required to achieve 50% protection of MDBK cells from BVDV-induced cytopathogenicity, as determined by the MTT method.

^g-h^ Compound concentration (μM) required to achieve 50% protection of BHK-21 cells from respectively YFV^(g)^ and Reo-1^(h)^ induced cytopathogenicity, as determined by the MTT method.

^i^ Compound concentration (μM) required to reduce the plaque number of HSV-1 by 50% in Vero-76 monolayers.

Surprisingly, 3-α-tigloyl-melianol (4) and melianone (5) showed a strong activity against YFV (EC_50_s values of 7 and 3 μM respectively), a representative of *Flavivirus* genus. Other relevant activities were not highlighted, with the exception of an anti-HSV-1 activity for compound 5 but at lower potency (EC_50_ = 20 μM). So, we decided to extend the screening to other two important human pathogens of *Flavivirus* genus, Dengue virus and West Nile virus. As showed in Table 2, 4 and 5 showed a very interesting activity against DENV-2 and WNV (with EC_50_s values in the range of 3–11 μM).

**Table 2:** Cytotoxicity and antiviral activity of 3-α-tigloyl-melianol and melianone against DENV-2 and WNV.

	*MTT assay*			*Plaque assay*	
Compounds	BHK-21	DENV-2	WNV	WNVd. i.	WNV2hrs p.i.
	^a^CC_50_	^b^EC_50_	^c^EC_50_	^d^EC_50_	^e^EC_50_
43-α-tigloylmelianol	20	3.0	3.0	3.5	>20
5melianone	50	12	11	6.1	>50
*Ref*. *compounds*					
2’-C-methyl-guanosine	80	1.4	1.0	1.0	1.0

Data represent mean values of three independent determinations. The variation among them was less than 15% respect to the mean value.

^a^ Compound concentration (μM) required to reduce the proliferation of mock-infected BHK-21 cells by 50%, as determined by the MTT method.

^b-c^ Compound concentration (μM) required to achieve 50% protection of BHK-21 cells from respectively DENV-2^(b)^ and WNV^(c)^ induced cytopathogenicity, as determined by the MTT method.

^d-e^ Compound concentration (μM) required to reduce the plaque number of WNV by 50% in BHK-21 monolayers, adding compounds during infection^(d)^ or 2 hours post infection^(e)^.

Despite the effort made in the last decade to understand their biology, many aspects such as the molecular interactions they use to enter cells and the identity of the cellular receptors involved in virus binding and internalization are far from being understood [33]. Particularly, we investigated the mode of inhibition against WNV, for which the effort for drug discovery is lower, compared with HCV or DENV, due to the perception that there is not an urgent need. With time, conversely, it is expected that the WNV seroprevalence will increase both in the human and bird populations [34].

**Fig 1:**
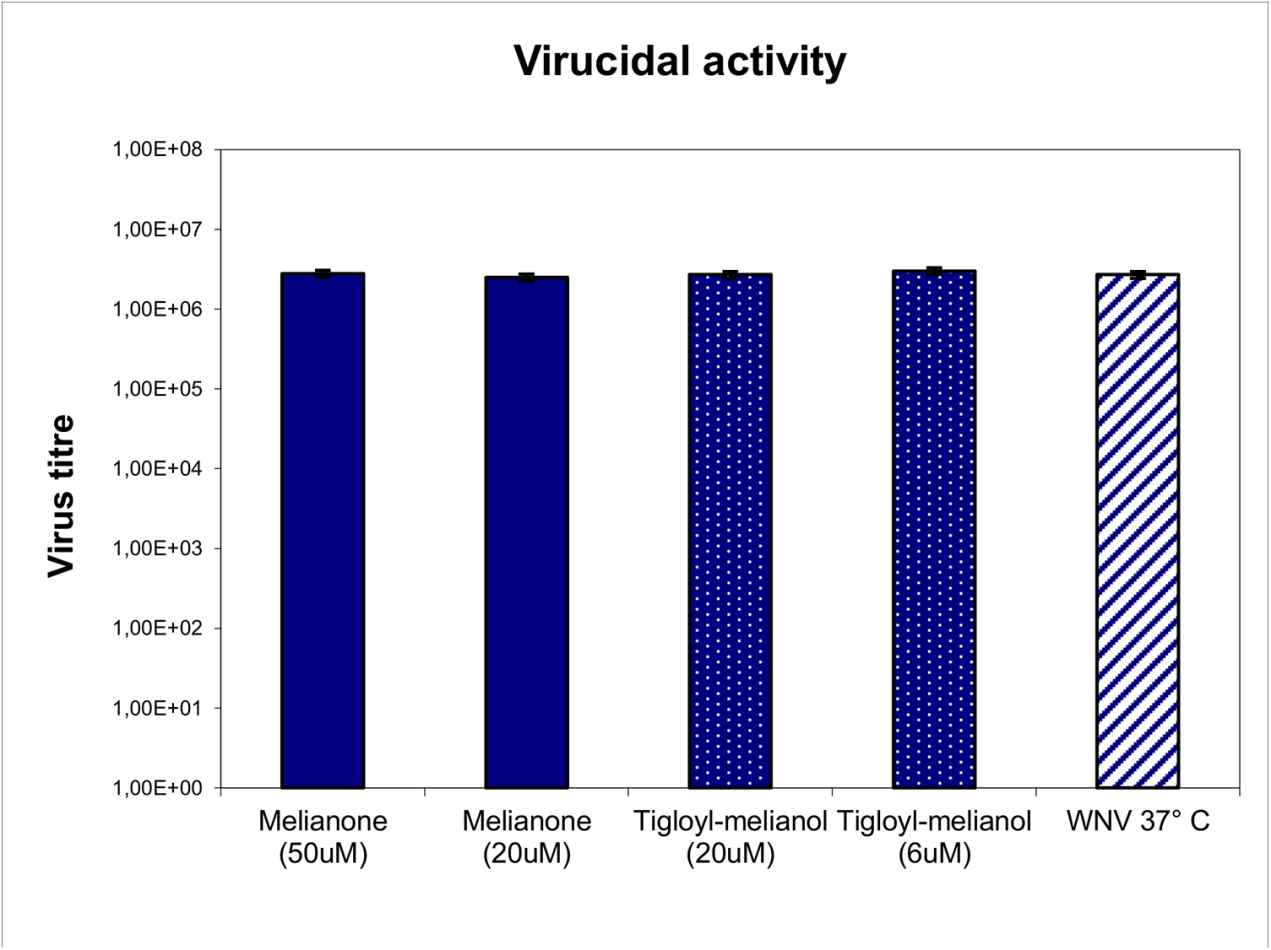
Virucidal activity of melianone and 3-α-tigloyl-melianol. Treatment at 37°C for 45 min. with melianone and 3-tigloyl-melianol at different concentrations doesn’t affect the WNV infectivity. Each value represents a mean of duplicate assays.

**Fig 2:**
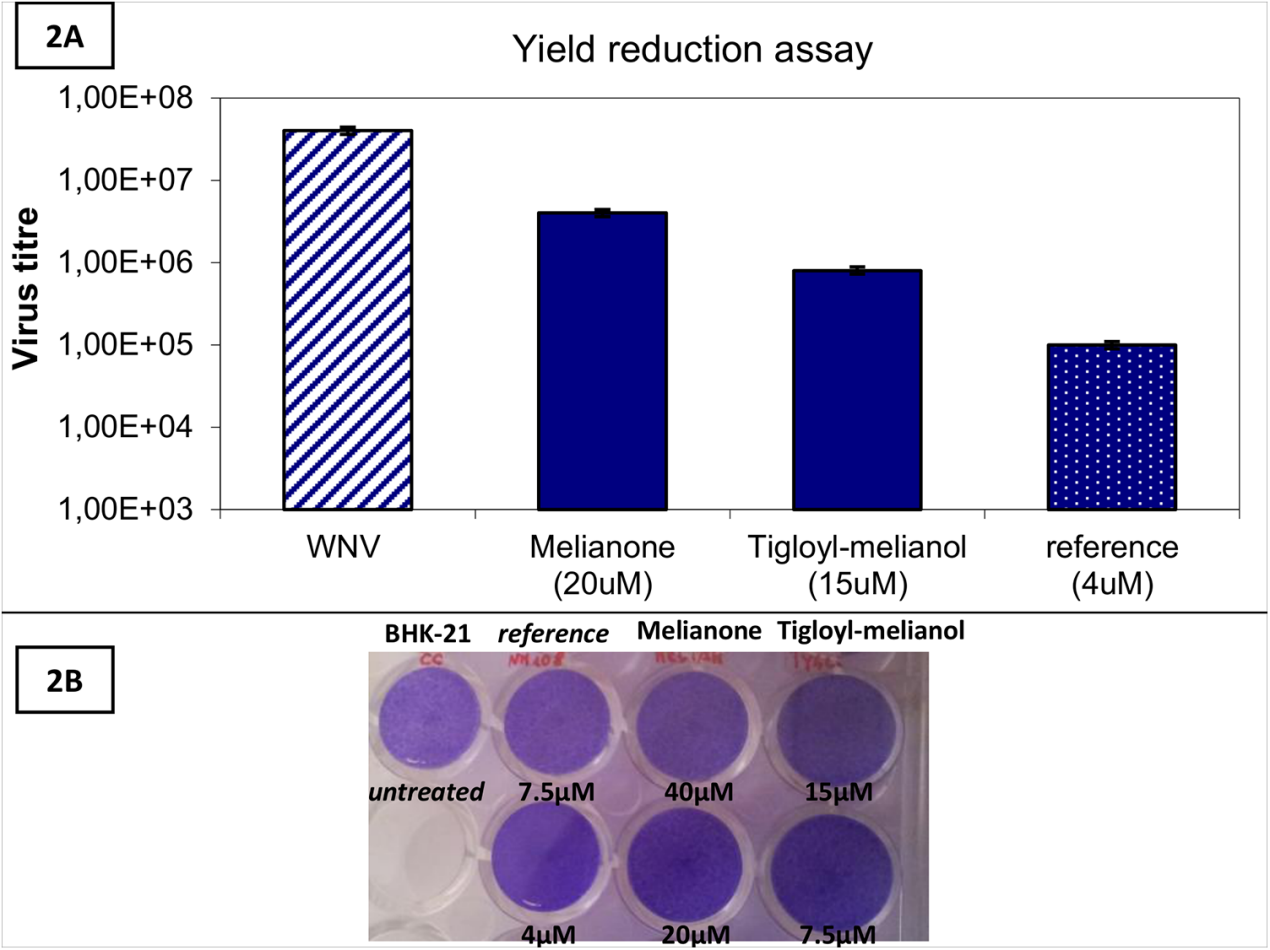
Yield reduction assay. **2A.** Reduction of WNV titre in presence of not cytotoxic concentrations of melianone (20μM) and 3-tigloyl-melianol (15μM). 2’-C-methylguanosine was used as reference compound (at 4μM). **2B.** The BHK-21 monolayers treated with the above compounds did not show significant cytopathic effects at the tested concentrations.

The potential virucidal activity of 4 and 5 was investigated, incubating a WNV solution containing 5x10^5^ PFU/ml for 45 min. at 37°C with two concentrations of each compound (6 and 20μM for 4; 20 and 50μM for 5). Tested compounds failed to affect the WNV infectivity (Fig 1), suggesting that the inhibition of WNV replication observed in cell-based assays is not due to the inactivation of virions, but can be totally attributed to an interference of compounds with the viral life cycle. The antiviral activity of 4 and 5 was further evaluated in an yield reduction assay, in order to demonstrate the reduction of virus titre in presence of two active compounds, during a single round of viral infection. Not cytotoxic concentrations of respectively 20 μM for 5 and 15μM for 4 were used; a reduction of virus titre was observed for both compounds (Fig 2).

**Fig 3:**
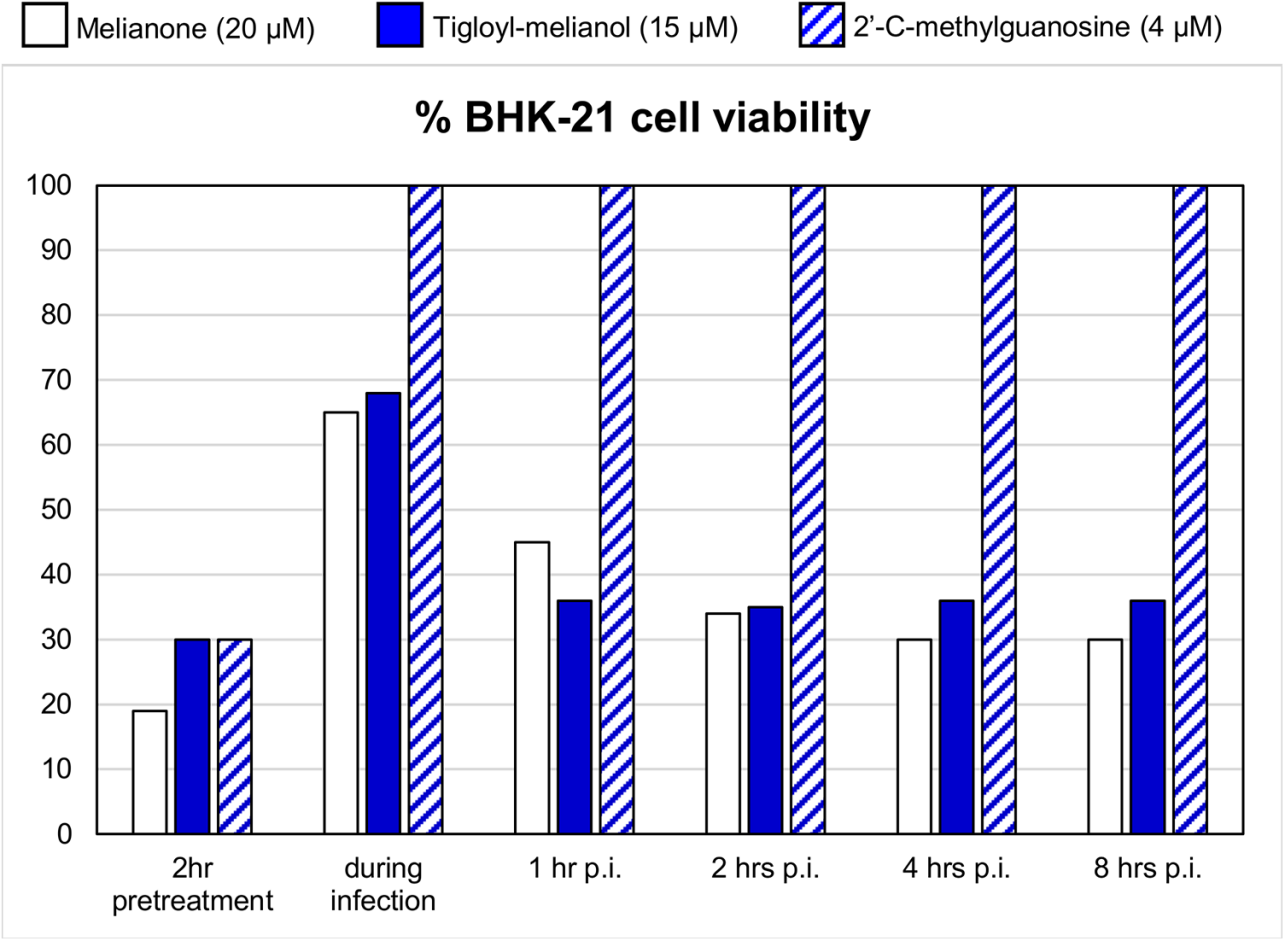
Time of drug addition. Melianone, 3-α-tigloyl-melianol and a reference compound were added during the infection and at different times post infection (p.i.). Each value represents a mean of duplicate assays. When added 1 hour or more p.i. the two compounds are not more able to protect BHK-21 cells, differently to reference compound. % of cell viability is reported; value for the untreated infected control is 15%.

To investigate the mechanism of 4 and 5 in inhibition of WNV, we performed a time of drug addition experiment to determine at what stage of life cycle the compounds inhibit (Fig 3). Concentrations of compounds were 15 μM for 4 and 20 μM for 5, while reference compound 2’-C-methylguanosine was added at 4 μM.

Pretreatment with 15μM of 4 or 20μM of 5 does not increase the cells viability when compared with infected control. The addition of compounds 4 and 5 during the infection increases the cell viability, as well as the reference compound. On the contrary, when added at 1, 2, 4 or 8 hours post-infection selected compounds are not more able to significantly increase the cell viability, compared to the untreated infected control and to the reference compound. These data suggest that both 3-α-tigloylmelianol and melianone inhibit WNV infection by targeting an early stage of the virus life cycle, probably during the first hour of infection.

Antiviral activity of 4 and 5 against WNV was also investigated in a plaque reduction assay, adding compounds both during the infection and 2 hours post-infection, in order to confirm the time-of-addition results. As shown in Table 2, EC_50_ values are similar to those found in MTT assay if compounds are added during the infection; activity was completely lost if compounds are added at 2 hours post infection.

**Fig 4:**
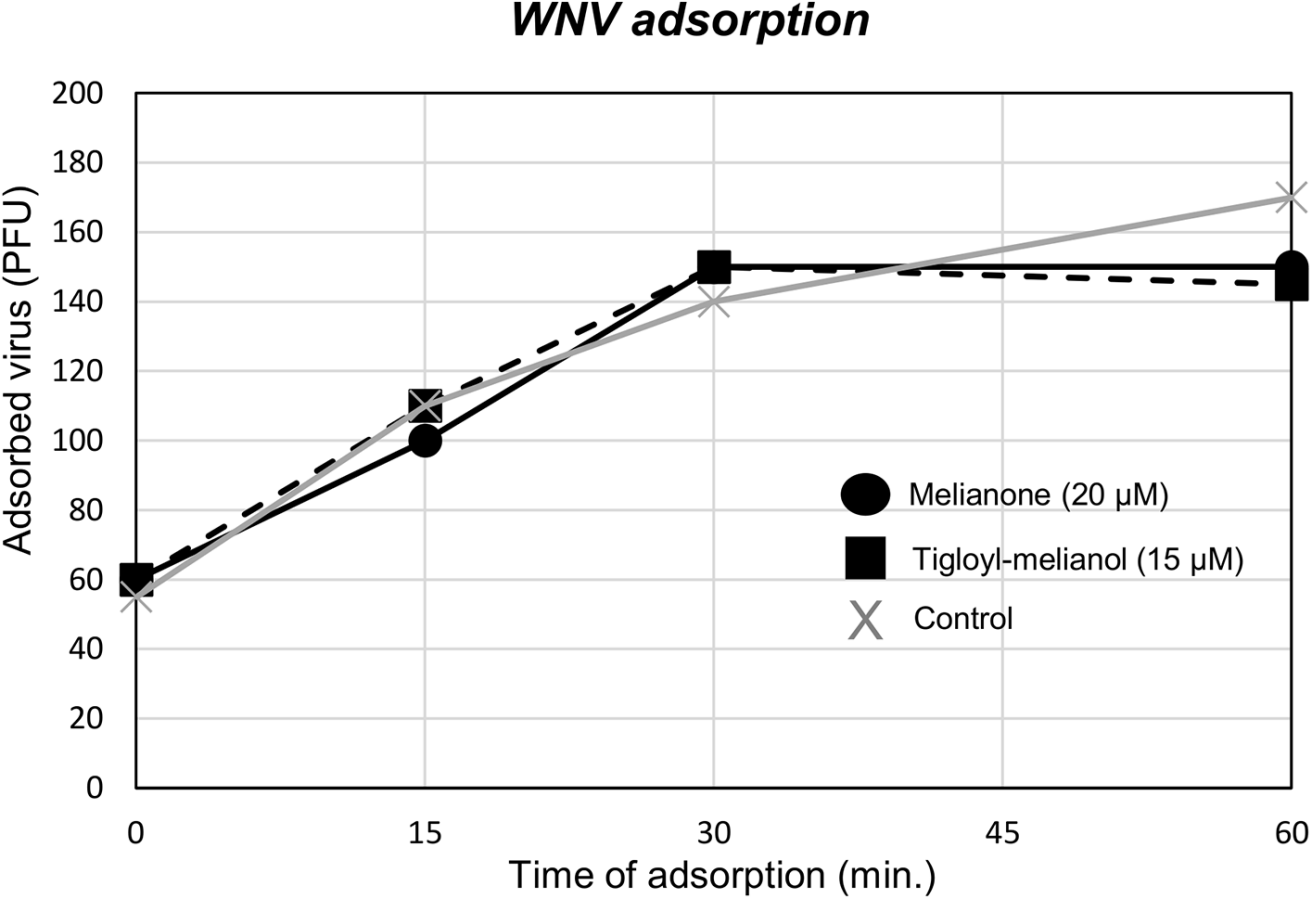
Effect of melianone and 3-α-tigloyl-melianol on WNV adsorption kinetic. BHK-21 cells were incubated during 0, 15, 30 and 60 min. at 4°C with WNV in the absence or presence of compounds. Each value represents a mean of duplicate assays. Treatment with both compounds doesn’t reduce the virus titre.

To better define the process of inhibition, the kinetics of virus adsorption in the presence of compounds was finally evaluated. As a matter of fact, low-temperature treatment allows binding of WNV to the cell surface receptors but prevents the internalization of virus particles into the cells [35,36]. BHK cells were incubated with WNV (m.o.i. = 1) and compounds for different times at 4°C, using 15μM for 4 and 20μM for 5. Results reported in Fig 4 show that the treatment with both compounds doesn’t reduce the virus titre in comparison to untreated infected control, suggesting that inhibition occurs after the adsoption step.

When we tested for antimycobacterial activity the limonoids from *M*. *azedarach* fruits, as showed in Table 3, we found that the most active of four tirucallane-type triterpenes was 3-*α*-tigloyl-melianol with a MIC value of 29 μM, followed by methyl kulonate exhibiting a MIC value of 70 μM; melianone was instead found not active up to 136 μM. Most importantly, 3-*α*-tigloyl-melianol was found not cytotoxic against Vero cells up to 500 μM, which fact makes it a valuable structure for further development into a potential antimycobacterial agent.

**Table 3:** Antimycobacterial activity (Minimum Inhibitory Concentration: MIC) of selected limonoids against *Mycobacterium tuberculosis*.

Compounds	MIC (μM)
2methyl kulonate	70
43-α-tigloylmelianol	29
5melianone	>136
*Ref*. *compound*	
Isoniazid	0.45

Data represent mean values of three independent determinations. The variation among them was less than 15% respect to the mean value.

## Conclusions

As discussed, various limonoids were found significantly active against different cancer cell lines. Although few limonoids have been described as antibacterials [37,38] and antivirals [18–23], this is the first report of antiviral limonoids from *M*. *azedarach*. We here demonstrate that two limonoids, 3-α-tigloyl-melianol and melianone, contained in the fruits are strongly active, at micromolar level, against three viruses belonging to *Flavivirus* genus, Dengue-2, West Nile virus and Yellow Fever virus. Their chemical structure could offer interesting indications for design and development of more potent derivatives, since that *Flavivirus* represent the most prevalent arthropod-borne viruses worldwide and there are currently no marketed drugs or clinical candidates for treatment or prevention of their infections in humans, that are continuously re-emerging and cause hemorrhagic fevers or neurological diseases.

The compounds do not prevent the recognition and the attachment to the cell receptors, but their mechanism of inhibition seems to be related to the following entry process or to an early event of life cycle, occurring in the first post-infection hour.

Furthermore, the most abundant limonoids of the *M*. *azedarach* fruits, 3-α-tigloyl-melianol and methyl kulonate, showed a significant activity against *M*. *tuberculosis*. Since the emergence of multi-drug resistance throughout the developing world, alternative methods of control must be used and 3-*α*-tigloyl-melianol and methyl kulonate could therefore be studied to that context for the treatment of *M*. *tuberculosis*. Additionally the lack of 3-*α*-tigloyl-melianol cytotoxicity increases its potential as natural antimycobacterial agent.
